# Over-stimulation of insulin/IGF-1 signaling by western diet may promote diseases of civilization: *lessons learnt from laron syndrome*

**DOI:** 10.1186/1743-7075-8-41

**Published:** 2011-06-24

**Authors:** Bodo C Melnik, Swen Malte John, Gerd Schmitz

**Affiliations:** 1 Department of Dermatology, Environmental Medicine and Health Theory, University of Osnabrück, Sedanstrasse 115, D-49090 Osnabrück, Germany; 2 Institute for Clinical Chemistry and Laboratory Medicine, University Clinic of Regensburg, Franz-Josef-Strauss-Allee 11, D-93053 Regensburg, Germany

## Abstract

The insulin/insulin-like growth factor-1 (IGF-1) pathway drives an evolutionarily conserved network that regulates lifespan and longevity. Individuals with Laron syndrome who carry mutations in the growth hormone receptor (*GHR*) gene that lead to severe congenital IGF-1 deficiency with decreased insulin/IGF-1 signaling (IIS) exhibit reduced prevalence rates of acne, diabetes and cancer. Western diet with high intake of hyperglycemic carbohydrates and insulinotropic dairy over-stimulates IIS. The reduction of IIS in Laron subjects unmasks the potential role of persistent hyperactive IIS mediated by Western diet in the development of diseases of civilization and offers a rational perspective for dietary adjustments with less insulinotropic diets like the Paleolithic diet.

## Introduction

**Figure 1:**
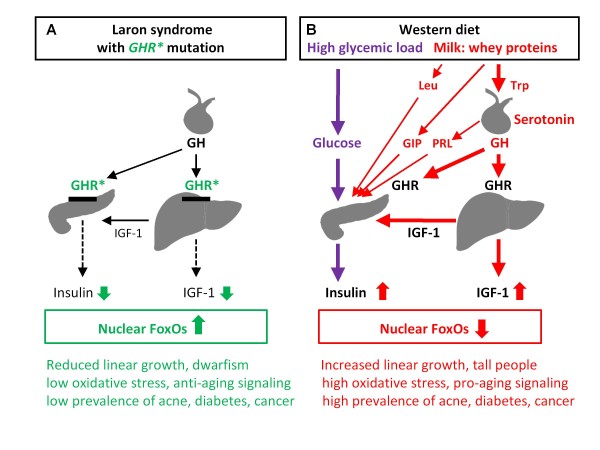
**Impact of insulin/IGF-1 signaling in Laron syndrome (A) and Western diet (B) on FoxO-mediated gene regulation and associated pathologies**. GHR*, growth hormone receptor loss of function mutation in Laron syndrome; GIP, glucose-dependent insulinotropic polypeptide, a whey protein-induced incretin, which stimulates β-cell proliferation and insulin secretion; PRL, prolactin; PRL secretion is induced by serotoninergic hypothalamic signaling; Trp, tryptophan, and Leu, leucine, essential amino acids enriched in the whey protein α-lactalbumin; Trp via serotonin synthesis stimulates pituitary GH and PRL secretion and Leu stimulates β-cell proliferation and insulin secretion.

Recently, Guevara-Aguirre *et al *reported on 99 Ecuadorian individuals with Laron syndrome due to growth hormone receptor (*GHR*) deficiency and congenital insulin-like growth factor-1 (IGF-1) deficiency who did not develop type 2 diabetes (T2D) and were almost free of cancer, in contrast to their healthy relatives with normal insulin/IGF-1 signaling (IIS) [1]. A recent worldwide survey of Steuerman *et al *demonstrated that none of 230 individuals with Laron syndrome developed cancer [2]. Laron syndrome is a very informative experiment of nature and uncovers the link between low IIS and the related protection from diseases of civilization in contrast to exaggerated IIS induced by Western diet as shown in Figure 1.

### Insulin/IGF-1/FoxO signaling in nonhuman organisms

Over 20 years ago it was discovered that mutations in *daf-2 *and *age-1 *double the lifespan in worms [3,4]. *Daf-2 *encodes the only insulin/IGF-1 receptor expressed in worms and *age-1 *is the catalytic subunit of the downstream phosphoinositide 3-kinase (PI3K). Substantial evidence has been provided that IIS is an evolutionarily conserved pathway that regulates lifespan across many species like flies, worms, and mice [3-6]. Decreased IIS in nonhuman organisms has been associated with extended lifespan and protection against oxidative stress-mediated age-dependent damage [5,6]. IIS activates PI3K and Akt kinase. Akt-mediated phosphorylation activates the kinase mammalian target of rapamycin complex 1 (mTORC1) activating its effector S6 kinase 1 (S6K1) involved in the up-regulation of protein synthesis and cell proliferation. Furthermore, activated Akt stimulates specific phosphorylation of FoxO proteins in the nucleus leading to their extrusion into the cytoplasm [5]. FoxO transcription factors have emerged as a convergence point of IIS, nutrient availability and oxidative stress [6]. Increased expression of DAF-16, the ortholog of human FoxO proteins in the worm *Caenorhabditis elegans*, due to a mutation of the insulin/IGF-1 receptor *daf-2 *has been found to significantly increase the worm's lifespan [2,3]. Male and female heterozygous IGF-1 receptor knockout mice *Igf1r*^*+/- *^live 16% and 33% longer than wild-type males and females, respectively [7]. Thus, convincing experimental evidence obtained from invertebrates and nonhuman vertebrates taught us that down-regulated IIS is of critical importance for metabolic homeostasis, improved oxidative stress responses and longevity.

The pathogenesis of age-related diseases has been associated with an impaired capacity to counteract cellular damage induced by oxidative stress. In T2D some of the consequences of an oxidative environment are the development of insulin resistance, β-cell dysfunction, impaired glucose tolerance, and mitochondrial dysfunction [8]. Oxidative stress, implicated in the etiology of cancer, results from an imbalance in the production of reactive oxygen species (ROS) and the cell's own antioxidant protection. ROS deregulate the redox homeostasis and promote tumor formation by initiating an aberrant induction of signaling networks that cause tumorigenesis [9]. FoxO proteins are pivotal regulators of oxidative stress resistance and activate the expression of manganese superoxide dismutase and catalase [5]. Moreover, FoxO1 at the promoter level induces expression of *Hmox1 *(heme oxygenase 1) thereby decreasing mitochondrial respiration and ROS formation [5]. Thus, increased IIS with down-regulated nuclear FoxO levels impairs adequate elimination of ROS, a critical mechanism involved in the promotion of acne, T2D and cancer.

### Insulin/IGF-1/FoxO signaling and type 2 diabetes

FoxO1 inhibits β-cell proliferation [10]. Nutritional alterations of β-cell FoxO1 transcriptional activity are predominantly mediated through glucose-stimulated insulin secretion and insulin receptor signaling. Recently, the concept of a "metabolic diapause" has been proposed for the changes induced by FoxO1 to protect β-cells against oxidative stress underpinning the concept of β-cell rest as a treatment goal in T2D [11]. Thus, FoxO1, the convergence point of IIS, orchestrates β-cell proliferation and apoptosis which both are increased in T2D [12].

### Insulin/IGF-1/FoxO signaling and cancer

GH, IGF-1 and insulin have cancer-promoting actions and raised serum IGF-1 levels have been associated with increased risk of prostate, breast and colorectal cancers [13]. Steuerman *et al *conducted a large worldwide survey on the prevalence of cancer in patients with various causes of secondary congenital IGF-1 deficiency and confirmed that subjects with Laron syndrome with congenital IGF-1 deficiency seem protected from the development of cancer [2]. IIS regulates the nuclear distribution of FoxO proteins which are increasingly considered to represent unique cellular targets directed against human cancer in light of their pro-apoptotic effects and their ability to lead to cell cycle arrest [5,14]. FoxOs are involved in the control of angiogenesis, stem cell proliferation, cell adhesion, oxidative stress responses, as well as innate and acquired immunity. Permanently increased IIS with consecutive down-regulation of nuclear FoxO levels may thus promote the development of cancer [14].

### Insulin/IGF-1/FoxO signaling and acne

Acne is epidemic in adolescents of Western life style and absent in populations consuming a *Paleolithic diet *excluding sugar, grains and dairy like the Kitava islanders who exhibit low basal insulin levels compared with age-matched Europeans [15]. Accumulating evidence supports the view that consumption of hyperglycemic carbohydrates and insulinotropic dairy are the driving force of the acne epidemic in Western countries [16]. IGF-1 is a strong stimulator of sebaceous lipogenesis and acne pathogenesis has been linked to elevated IIS with consecutive nuclear FoxO deficiency [16,17]. Moreover, IGF-1 stimulates androgen receptor signaling by increasing adrenal and gonadal androgen synthesis as well as androgen receptor transactivation by nuclear extrusion of the androgen receptor cosuppressor FoxO1 [17,18]. Thus, acne can be considered as a visible model disease of exaggerated IIS during puberty aggravated by Western diet, just the opposite of deficient IIS in untreated Laron subjects who do not develop acne [19]. On the other hand, seborrhea, acne, T2D and cancer are frequently observed in acromegaly with increased GH/IGF-1 signaling in comparison to healthy controls [16]. Intriguingly, an association between the prevalence of severe tetracycline-treated acne and increased risk of prostate cancer later in life has been observed [20].

### Western diet potentiates insulin/IGF-1 signaling

Grain-based food, sugars and dairy products are the nutritional staples of Western diet. High glycemic load diets have been recognized as aggravating factors of acne, whereas a low glycemic load diet improved acne and decreased the bioavailability of free IGF-1 in plasma [21].

Milk and fermented dairy products containing whey proteins exhibit a high *insulinemic index *in comparison to their low carbohydrate content [22]. This phenomenon appears to be the secret of IIS of mammalian milk, an evolutionarily provided program for the promotion of neonatal growth [16]. Cow milk consumption in humans shifts the somatotropic axis to higher levels and significantly increases serum levels of GH and IGF-1 [23] (Figure 1B). Oral ingestion of the whey protein α-lactalbumin has been shown to increase the somatotropic axis in healthy women. There is strong epidemiological evidence that dairy consumption significantly increases serum IGF-1 levels in humans [24]. This explains why high intake of milk increases linear growth [25]. In contrast, short stature is a characteristic feature of congenital IGF-1 deficiency in Laron syndrome. Thus, Western diet shifts the GH/IGF-1 axis to abnormally high levels, just in the opposite direction of low IIS observed in Laron syndrome [1,2] (Figure 1).

### Exaggerated insulin/IGF-1 signaling by Western diet and type 2 diabetes

Insulin resistance and hyperinsulinemia are characteristic features of the metabolic syndrome. Extensive consumption of hyperglycemic food with increased glucose-mediated signal transduction to pancreatic β-cells is a major factor of glucose/FoxO1-mediated β-cell proliferation and impaired β-cell oxidative stress responses [8-11].

Milk consumption after the weaning period maintains high levels of IIS persistently stimulating pancreatic β-cell proliferation [26]. Continued over-stimulation of pancreatic β-cells by whey protein-driven IIS after the post-weaning period may continuously diminish nuclear FoxO levels, thereby promoting oxidative stress damage of β-cells finally resulting in early onset of β-cell cellular senescence and apoptosis [26]. Indeed, increased proliferation and apoptosis of β-cells during lifetime are hallmarks of T2D [12].

### Exaggerated insulin/IGF-1 signaling of Western diet and cancer

Increased IIS has been implicated to play an important role in most types of epithelial neoplasia [13,27]. Higher serum IGF-1 levels were associated with increased risk of cancer death in older community-dwelling men [28]. On the other hand, subjects with Laron syndrome exemplify that low IIS is associated with a low prevalence of cancer [1,2]. However, it should be mentioned that Laron subjects are not longer lived than normal subjects eating a Western diet and if not properly treated die of cardiovascular diseases.

## Conclusion and future perspectives

Laron syndrome with decreased IIS is associated with a reduced prevalence of acne, T2D and cancer. In contrast, up-regulated IIS by Western diet appears to promote the development of chronic diseases of civilization. Paleolithic diet, which excludes hyperglycemic carbohydrates and insulinotropic dairy, has been successfully introduced for the prevention and treatment of acne, T2D and cardiovascular diseases [16,29]. Future efforts should be undertaken to lower the high insulinemic index of milk (I.I. 140) and other whey-based milk products to reach values of beef (I.I. 51) or cheese (I.I. 45) [16,29]. Furthermore, combinations of hyperglycemic carbohydrates and insulinotropic dairy with potentiating effects on IIS should be restricted.

Individuals with genetic single nucleotide polymorphisms (SNPs) resulting in hyperactive IIS may be at special risk for the development of age-related diseases, predominantly when their high intrinsic IIS is superposed by exaggerated IIS of Western diet. Intriguingly, genetic variations with reduced IIS due to SNPs of interacting components of the IIS cascades (*GH1, IGF1, IGF1R, IRS1, FoxO1A, FoxO3A*) and have been associated with increased longevity [30,31]. Thus, future research should consider the impact of interacting intrinsic genetic as well as extrinsic dietary factors involved in the regulation of IIS.

The access to higher amounts of insulinotropic and IGF-1-raising foods (sugar, grains and dairy) occurred about 10,000 years ago during the *Neolithic Revolution *and was further augmented by the *Industrial Revolution*. However, the human genome may not have adapted to this "recent switch" to higher IIS driven by Western diet. According to mitochondrial DNA data, modern humans with nearly similar genomic structure lived roughly 200,000 years ago and consumed a less insulinotropic Paleolithic diet. In this regards, it has been proposed to re-adapt our nutrition to the beneficial characteristics of our pre-agricultural diets [32]. The time point introducing a well-balanced Paleolithic diet may be a special issue of concern as proper IIS is important for the function of the reproductive and central nervous system. In adulthood however, dietary restrictions decreasing IIS may reduce the risk of age-associated pathology like proteotoxicity as recently demonstrated in a mouse model of Alzheimer disease [33].

## Abbreviations

Age-1: catalytic subunit of PI3K of *C. elegans*; Akt: Akt kinase (protein kinase B); *C. elegans*: *Caenorhabditis elegans*; DAF-2: Insulin/IGF-1 receptor of *C. elegans*; DAF-16: dauer form 16 (FoxO ortholog in the worm); FoxO: forkhead box transcription factor class O; GH: growth hormone; GHR: growth hormone receptor; G.I.: glycemic index; GIP: glucose-dependent insulinotropic polypeptide (gastric inhibitory polypeptide); I.I.: insulinemic index; IIS: insulin/IGF-1 signaling; IGF-1: insulin-like growth factor-1; IGF1R: insulin-like growth factor-1 receptor; IR: insulin receptor; IRS: insulin receptor substrate; PI3K: phosphoinositide-3 kinase; Leu: leucine; mTORC1; mammalian target of rapamycin complex 1; ROS: reactive oxygen species; S6K1: S6 kinase 1; T2D: type 2 diabetes; Trp: tryptophan.

## Competing interests

The authors declare that they have no competing interests.

## Authors' contributions

BCM and GS researched data, contributed to discussion and conclusion. SMJ contributed to conclusion and reviewed/edited manuscript. All authors read and approved the final manuscript.
